# Examination of Physiological Function and Biochemical Disorders in a Rat Model of Prolonged Asphyxia-Induced Cardiac Arrest followed by Cardio Pulmonary Bypass Resuscitation

**DOI:** 10.1371/journal.pone.0112012

**Published:** 2014-11-10

**Authors:** Junhwan Kim, Tai Yin, Ming Yin, Wei Zhang, Koichiro Shinozaki, Mary A. Selak, Kirk L. Pappan, Joshua W. Lampe, Lance B. Becker

**Affiliations:** 1 Center for Resuscitation Science, Department of Emergency Medicine, University of Pennsylvania, Philadelphia, Pennsylvania, United States of America; 2 Metabolon Inc., Durham, North Carolina, United States of America; Azienda Ospedaliero-Universitaria Careggi, Italy

## Abstract

**Background:**

Cardiac arrest induces whole body ischemia, which causes damage to multiple organs particularly the heart and the brain. There is clinical and preclinical evidence that neurological injury is responsible for high mortality and morbidity of patients even after successful cardiopulmonary resuscitation. A better understanding of the metabolic alterations in the brain during ischemia will enable the development of better targeted resuscitation protocols that repair the ischemic damage and minimize the additional damage caused by reperfusion.

**Method:**

A validated whole body model of rodent arrest followed by resuscitation was utilized; animals were randomized into three groups: control, 30 minute asphyxial arrest, or 30 minutes asphyxial arrest followed by 60 min cardiopulmonary bypass (CPB) resuscitation. Blood gases and hemodynamics were monitored during the procedures. An untargeted metabolic survey of heart and brain tissues following cardiac arrest and after CPB resuscitation was conducted to better define the alterations associated with each condition.

**Results:**

After 30 min cardiac arrest and 60 min CPB, the rats exhibited no observable brain function and weakened heart function in a physiological assessment. Heart and brain tissues harvested following 30 min ischemia had significant changes in the concentration of metabolites in lipid and carbohydrate metabolism. In addition, the brain had increased lysophospholipid content. CPB resuscitation significantly normalized metabolite concentrations in the heart tissue, but not in the brain tissue.

**Conclusion:**

The observation that metabolic alterations are seen primarily during cardiac arrest suggests that the events of ischemia are the major cause of neurological damage in our rat model of asphyxia-CPB resuscitation. Impaired glycolysis and increased lysophospholipids observed only in the brain suggest that altered energy metabolism and phospholipid degradation may be a central mechanism in unresuscitatable brain damage.

## Introduction

Cardiac arrest is one of the leading causes of death affecting over 300,000 people each year in the US [1]. Cardiac arrest induces whole body ischemia, which causes critical damage to multiple organs, including the heart and the brain [2]. Rapid resuscitation will successfully treat most cardiac arrest patients, but with each passing minute of ischemia, the likelihood of survival dramatically decreases. Even after achieving return of spontaneous circulation (ROSC), survival rate is still lower than 50% [3]. This poor survival is mainly caused by cerebral dysfunction [4]–[6].

To improve survival rates, various controlled resuscitation methods have been tested in animal models and in clinical studies [7], [8]. Cardiopulmonary bypass (CPB) is an emerging method for controlled resuscitation of patients with cardiac arrest. Many medical centers worldwide, including in the US, have shown that CPB successfully resuscitates patients who do not respond to conventional CPR [8]–[11]. Successful resuscitation requires repairing the defects caused by ischemia while minimizing additional damage provoked by reperfusion, which is dependent on the disordered cellular conditions generated during the preceding ischemia [12]. Therefore, a better understanding of the metabolic and biochemical status of ischemic tissue will provide therapeutic targets for the development of more effective resuscitation strategies.

Prior studies of post-ischemic dysfunction in cells and isolated organs have described important cellular disorders, such as ATP depletion, altered calcium and other ion gradients, and altered lipid/membrane function [13], [14]. Particularly, phospholiphase A2-mediated decomposition of membrane phospholipids is thought to be responsible for irreversible tissue damage [15]. However, the link between observed metabolic alternations and organ function is poorly understood.

In this study, rats are subjected to 30 min asphyxia-induced cardiac arrest followed by CPB resuscitation. The system creates a reproducible ischemic injury [16], [17] and provides reliable achievement of ROSC even after prolonged cardiac arrest. Using asphyxia-CPB, we examined the effect of 30 min cardiac arrest on the metabolic and biochemical alterations in the heart and the brain and how metabolism further changes following reperfusion. Particularly, differences in the metabolic profiles of the heart and brain tissues are highlighted to elucidate possible mechanisms of unresuscitatable brain damage. Tissue specific metabolic results are compared to tissue specific functional results that were measured during the experiment.

## Materials and Methods

### Animals and chemicals

The experimental protocol for the study was approved by the Institutional Animal Care and Use Committee of the University of Pennsylvania (protocol number 803328). Adult male Sprague–Dawley rats (weight 420–470 g, Charles River Production, Wilmington, MA, USA), housed in a rodent facility under 12 h light–dark cycle with unrestricted access to food and water, were used for the study. Chemicals used for this study were purchased from major chemical suppliers.

### Asphyxia and cardiopulmonary bypass

The detailed procedures were published elsewhere [18]. Briefly, rats were anesthetized with 1–2% isoflurane and mechanically ventilated to maintain an EtCO_2_ between 35 and 45 mmHg. The left femoral artery and vein were separately cannulated for arterial and central venous pressure measurement. The right external jugular vein and the right femoral artery were cannulated for the venous outflow and arterial inflow ports. After surgical preparation, heparin (150 U) and vecuronium bromide (2 mg/kg IV) were administered and isoflurane was discontinued. Asphyxial cardiac arrest was induced by switching off the ventilator for 30 minutes. Mean arterial pressure below 20 mmHg was defined as cardiac arrest [18].

After 30 min of asphyxia, resuscitation was started with the initiation of CPB flow, which lasted 60 min. The customized CPB circuit designed for rodents consisted of a heat exchanger, an open venous reservoir, a membrane oxygenator, silicone tubing and a roller pump. For the oxygenator, 100% oxygen was used with a flow rate of 200 mL/min. Twenty mL of prime fluid and 20 mL of additional fluid (P-lyte) was added to the venous reservoir; the initial CPB flow rate was 70 mL/min and gradually decreased to ∼20 mL/min to meet venous outflow. With initiation of CPB, ventilation was resumed and subsequently adjusted to a PaCO_2_ of 35 to 45 mm Hg. Rats were sacrificed by decapitation either following 30 min cardiac arrest or 30 min cardiac arrest followed by 60 min resuscitation. Control rats were decapitated 7 min after administration of isoflurane. During the procedures, blood gas and hemodynamics were monitored.

### Metabolomics analysis

Heart and brain cortex, harvested from control, post cardiac arrest, or post resuscitation rats, were pulverized in liquid nitrogen and stored at −80°C until a complete set of samples were collected. Five samples in each group were sent to Metabolon Inc. (Durham, NC, USA) for metabolomics analysis [19], [20]. Tissue samples were extracted and prepared for analysis using Metabolon' standard solvent extraction method. The extracted samples were split into equal parts for analysis on the GC-MS and LC-MS/MS. For GC-MS, samples were derivatized using bistrimethyl-silyl-triflouroacetamide and analyzed on a Thermo-Finnigan Trace DSQ fast-scanning single-quadrupole mass spectrometer. LC-MS and MS/MS analysis was performed on a Thermo-Finnigan LTQ mass spectrometer. Data are presented as mean ± standard error of the mean unless otherwise stated. Welch's two-sample t-test was used to determine the significance of the difference.

## Results

### Blood gas

Following 30 min of asphyxia, blood pH was decreased from 7.43 to 7.00 and CO_2_ pressure (PCO_2_) was increased from 35.0 to 103.4 mmHg (Table 1). Oxygen concentration (PO_2_) was decreased from 98.7 to 11.3 mmHg and the saturation level of oxygen in hemoglobin (SO_2_) was also decreased from 96.9 to 7.0%. HCO_3_ concentration and hematocrit level did not change. The concentration of lactate was increased from 0.99 to 9.6 mmol/L, indicating increased anaerobic metabolism. Overall, these data showed that 30 min of asphyxia generates respiratory acidosis and hypoxia, which are typical phenomena of ischemia [21]. Following CPB resuscitation, pH, SO_2_, and PCO_2_ returned to the control levels (Table 1). The higher PO_2_ after resuscitation was due to the ventilation with 100% O_2_ gas. Low hematocrit level was caused by a dilution of blood during bypass resuscitation. A previous study showed that oxygen delivery should be sufficient to meet the whole-body oxygen demand at this degree of anemia [22].

**Table 1:** Blood gas analysis, hematocrit, and lactate levels at baseline, 30 min post cardiac arrest, and 60 min post CPB (mean ± standard deviation, n = 5).

	Initial	CA30	CA+CPB
pH	7.43±0.04	7.00±0.04	7.35±0.24
PCO_2_ (mmHg)	35.0±5.2	103.4±20.1	34.1±9.3
PO_2_ (mmHg)	98.7±34.9	11.3±6.7	285.3±130.4
SO_2_ (%)	96.9±2.3	7.0±6.7	99.8±0.4
HCO_3_^-^ (mmol/L)	24.2±4.1	25.0±2.5	20.9±9.1
HCT (%)	40.8±4.4	41.4±6.3	18.8±4.5
Lactate (mmol/L)	1.0±0.2	9.6±0.5	7.9±1.7

### Cardiac function

Table 2 summarizes cardiac data on heart function. Within the first 3 min of asphyxia, all rats had a mean arterial pressure below 20 mmHg, our definition of cardiac arrest. Within 5 min of onset of asphyxia, heart rate, pulse pressure, and respiratory rates were essentially zero and this state continued for the remaining period of asphixa, confirming that rats are in cardiac arrest for this time period.

**Table 2:** Cardiac data during asphyxia and resuscitation (mean ± standard deviation, n = 5).

	initial	CA		CPB				
	0	5	30	5	10	20	40	60
HR^1^	319.5	∼0	∼0	297.6	243.3	240.3	241.3	236.8
	±20.6			±62.6	±82.0	±49.8	±87.1	±85.1
MAP^2^	69.4	∼0	∼0	51.5	50.3	48.9	51.0	55.6
	±24.9			±8.7	±11.5	±6.3	±12.0	±13.9
Pulse Pressure	37.0	∼0	∼0	8.6	18.5	12.7	14.5	23.3
	±9.0			±13.5	±13.0	±7.4	±8.7	±12.8
Respiratory Rate	43.2	∼0	∼0	27.6	35.3	38.0	39.2	39.8
	±3.1			±9.7	±8.0	±6.9	±7.3	±6.8

1 HR, heart rate;

2 MAP, mean arterial pressure.

Table 2 also shows that CPB substantially restored cardiac function, with an average ROSC time of ∼8 min. Mean arterial pressure instantly increased to 30–40 mmHg upon CPB bypass and further increased to over 50 mmHg after ROSC. Heart rate also recovered significantly within the first 5 min after ROSC. Pulse pressure gradually increased during the 60 min CPB. However, it is noteworthy that some degree of hemodynamic compromise was still present after 60 min resuscitation; heart rate, mean artery pressure, and pulse pressure were 74, 80, and 62% of the initial rates, respectively.

In addition, rats did not respond to neurological stimuli such as toe pinching or corneal reflexes during the bypass period. This observation suggests a lack of brain function in these rats. Urination, which had stopped during cardiac arrest, resumed during resuscitation, indicating that the kidneys had adequate function following 30 min cardiac arrest with CPB.

### Metabolomics Analysis

Metabolic profiles in heart and brain tissues following cardiac arrest and CPB resuscitation were analyzed by mass spectrometry. Global metabolomics analysis identified ∼300 known small molecules in heart tissue and ∼250 in brain tissue. These molecules include amino acids, peptides, carbohydrates, lipids, nucleotides, cofactors, vitamins, and xenobiotics. Changes in the relative amounts of these metabolites following cardiac arrest and resuscitation were compared to their control levels. Tables 3 and 4 show the 38 metabolites with the greatest fold change, either increased or decreased.

**Table 3:** Metabolic changes in heart tissue (n = 5; ***bold italic***, p<0.05; *italic*, 0.05<p<0.1).

	metabolism^1^	CA/con.	CPB/con.
Increased			
mannitol	C	***12.82***	***15.40***
3-hydroxybutyryl CoA	F	***8.59***	1.01
valerylcarnitine	L	***7.31***	0.76
adenosine 5'diphosphoribose	F	6.61	***6.60***
3-hydroxybutyrate	L	***5.36***	***2.09***
hydroxybutyrylcarnitine	L	***5.31***	***1.91***
xanthosine	N	***3.77***	*1.36*
4-hydroxybutyrate	L	***3.76***	1.57
ribitol	C	***3.44***	***2.98***
phenylcarnitine	X	***2.69***	***3.19***
coenzyme A	F	*2.58*	0.66
threonylisoleucine	P	2.43	0.70
1,2-propanediol	L	2.41	***3.07***
2-methylbutyrylcarnitine	A	***2.39***	*1.59*
acetyl CoA	F	*2.30*	*0.68*
sorbitol	C	*2.29*	***6.92***
hippurate	X	***2.25***	***7.12***
adenine	N	***2.11***	***0.76***
propionylcarnitine	L	***2.10***	1.02
Decreased			
glucose-6-phosphate (G6P)	C	***0.04***	***0.37***
maltotriose	C	***0.05***	*0.49*
maltopentaose	C	***0.05***	***0.22***
fructose-6-phosphate	C	***0.05***	***0.44***
mannose-6-phosphate	C	***0.06***	***0.34***
glucose 1-phosphate	C	***0.09***	***0.49***
maltotetraose	C	***0.10***	*0.35*
glucose	C	***0.13***	***0.49***
oleoylcarnitine	L	***0.17***	***0.34***
chenodeoxycholate	L	***0.20***	***0.14***
sedoheptulose-7-phosphate	C	***0.21***	*0.64*
mannose	C	***0.23***	***0.46***
alpha-muricholate	L	***0.23***	***0.27***
aspartate	A	***0.26***	***0.56***
mannose-1-phosphate	C	***0.33***	***0.63***
2-stearoylglycerophosphocholine	L	0.33	0.76
1-stearoylglycerophosphoethanolamine	L	*0.34*	0.80
beta-muricholate	L	*0.38*	***0.26***
maltohexaose	C	*0.40*	0.45

1 C, carbohydrate; P, peptide: A, Amino acid; L, lipid; N, nucleotide; X, xenobiotics; O, organic acid; F, cofactor

**Table 4:** Metabolic changes in brain tissue (n = 5; ***bold italic***, p<0.05; *italic*, 0.05<p<0.1).

	metabolism^1^	CA/con.	CPB/con.
increased			
2-arachidonoylglycerophosphocholine	L	***8.13***	***8.24***
2-palmitoylglycerophosphocholine	L	***7.55***	***8.74***
2-docosahexaenoylglycerophosphocholine	L	***6.44***	***7.42***
2-oleoylglycerophosphocholine	L	***6.43***	***8.08***
1-arachidonoylglycerophosphocholine	L	***5.95***	***7.99***
xylitol	L	***5.94***	***8.02***
1-docosahexaenoylglycerophosphocholine	L	***4.78***	*5.75*
sphingosine	L	***4.29***	***5.23***
2-palmitoylglycerophosphoethanolamine	L	***4.25***	*4.10*
hydroxybutyrylcarnitine	L	***4.08***	***4.18***
3-hydroxybutyrate	L	***4.06***	***3.69***
1-oleoylglycerophosphocholine	L	***4.02***	*5.99*
2-docosapentaenoyl glycerophosphoethanolamine	L	***3.79***	*3.92*
2-arachidonoylglycerophosphoethanolamine	L	***3.76***	*3.23*
mannitol	C	***3.64***	***7.22***
1-palmitoylglycerophosphocholine	L	***3.64***	*4.47*
butyrylcarnitine	L	***3.59***	*2.07*
palmitoylcarnitine	L	***3.50***	*5.60*
sorbitol	C	***3.49***	***4.30***
decreased			
glucose	C	***0.11***	*0.43*
6-phosphogluconate	C	***0.21***	***0.38***
acetylcholine	L	***0.25***	0.78
glutamine-leucine	P	***0.32***	0.61
S-lactoylglutathione	A	***0.37***	***0.41***
guanosine	N	***0.43***	***0.57***
glucose-6-phosphate (G6P)	C	0.50	0.52
spermidine	A	***0.51***	0.72
adenosine 5'-monophosphate	N	***0.51***	*0.75*
citrate	O	***0.58***	1.37
3-dehydrocarnitine	L	*0.58*	0.63
sedoheptulose-7-phosphate	C	***0.59***	***0.51***
lathosterol	L	*0.59*	0.77
mannose-6-phosphate	C	0.64	0.62
ribulose 5-phosphate, xylulose 5-phosphate	C	***0.67***	***0.51***
adenosine	N	***0.68***	0.89
benzoate	X	***0.69***	0.85
pyrophosphate	E	0.70	0.97
lidocaine	X	0.70	0.87

1 C, carbohydrate; P, peptide: A, Amino acid; L, lipid; N, nucleotide; X, xenobiotics; O, organic acid

Following cardiac arrest, short-chain acyl carnitines (valerylcarnitine, hydroxybutyrylcarnitine, 2-methylbutyrylcarnitine, and propionylcarnitine) and 3-hydroxybutyryl CoA were significantly increased in heart tissue. Short-chain acyl carnitines and CoAs are derived from fatty acids or, in some cases from branched-chain amino acids, and represent incomplete mitochondrial oxidation products. Accumulation of these CoAs and carnitines are expected during ischemia-induced hypoxia due to the lack of oxygen to support operation of the mitochondrial oxidative phosphorylation [23]. However, oleoylcarnitine was decreased following cardiac arrest (Table 3), suggesting that long-chain fatty acids may have been continued to be partially metabolized through the mitochondrial beta-oxidation pathway or that their import into the mitochondria via carnitine-palmitoyl transferase-mediated transport was halted during ischemia as reported previously [24]. Alternatively, long-chain fatty acids have been metabolized for the synthesis of phospholipids during ischemia in the heart.

Another group of molecules accumulated during cardiac arrest was organic osmolytes, such as mannitol, ribitol, and sorbitol (Table 3). Organic osmolytes are known to accumulate in tissues in response to the hyperosmolarity caused by the increased concentrations of inorganic ions [25]. Since ischemia increases intracellular concentration of Ca^2+^, Na^+^, and K^+^ ions [26], the increase in the organic osmolytes is also expected in ischemic tissues. The protective roles of the organic osmolytes are well reviewed elsewhere [25].

Carbohydrate metabolites were substantially decreased in the heart tissue, particularly metabolites in the glycolysis pathway (glucose, glucose-6-phosphate, and fructose-6-phosphate), the pentose phosphate pathway (sedoheptulose 7-phosphate), and their precursors (mannose-6-phosphate and glucose-1-phosphate). Maltotriose, maltotetraose, maltopentaose, and maltohexaose (oligomeric forms of glucose) were also substantially decreased (Table 3).These data indicate that the heart continued to consume carbohydrates for energy generation during ischemia as previously discussed [27].

The metabolic and biochemical state of the heart tissues returned toward normal following 60 min CPB resuscitation; acyl carnitines and CoAs were decreased almost to the control level and carbohydrates were considerably increased compared to the cardiac arrest group. This observation is consistent with a resumption of cardiac function. However, the unchanged amounts of organic osmolytes suggest that more prolonged ionic or osmotic imbalances still existed following CPB.

In brain tissue following cardiac arrest, the most remarkable metabolic change was an increase in lysophospholipids, particularly lysophosphatidylethanolamine and lysophosphatidylcholine species (Table 4). The tissue also had increased amounts of short-chain acyl carnitines (hydroxybutyrylcarnitine and butyrylcarnitine) and organic osmolytes (xylitol, mannitol, and sorbitol) following cardiac arrest.

The glucose level was decreased 90% relative to controls in brain tissue following cardiac arrest. However, other carbohydrates were not decreased as much as was observed in heart tissue; glucose-6-phosphate, sedoheptulose 7-phosphate, and mannose-6-phosphate were decreased by less than 50%. Changes in metabolites associated with glycolysis are examined in more detail below. Unlike the heart tissue, the metabolic disorder found in brain tissue following cardiac arrest remained fixed and did not improve toward normal levels following CPB resuscitation (Table 4). Particularly, the concentration of lysophospholipids was not changed following resuscitation.

### Metabolites in glycolysis

**Figure 1:**
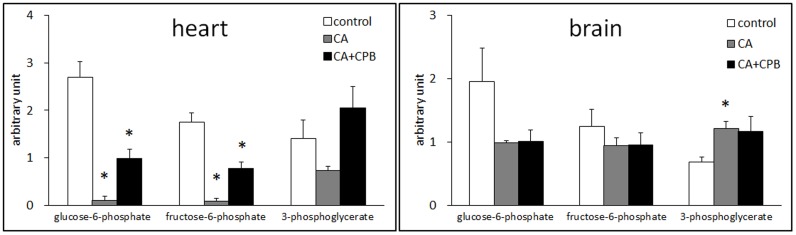
Changes in the relative amounts of glucose 6-phosphate, fructose-6-phosphate, and 3-phosholgycerate in the heart (left) and the brain (right) after 30 min cardiac arrest and 30 min cardiac arrest followed by 60 min CPB (n = 5).

Anaerobic glycolysis is the primary ATP generation pathway in the heart and the brain during ischemia. The levels of three intermediates in glycolysis, glucose-6-phosphate, fructose-6-phosphate, and 3-phosphoglycerate, were compared to assess the efficiency of glycolysis in these tissues. Glucose-6-phosphate and fructose-6-phosphate are intermediates in the early stage and 3-phosphoglycerate is in the late stage of glycolysis. Figure 1 shows that in heart tissue glucose-6-phosphate and fructose-6-phosphate were decreased by ∼95% and 3-phosphoglycerate was decreased by 47% compared to the control level following 30 min cardiac arrest. Following resuscitation, the concentration of glucose-6-phosphate and fructose-6-phosphate was increased to ∼40% of the control level and 3-phosphoglycerate was increased to the control level.

In brain, glucose-6-phosphate was decreased by 50% and fructose-6-phosphate by 36% following cardiac arrest, whereas 3-phosphoglycerate was increased by 76%. Interestingly, the relative contents of these compounds were not changed with CPB. These data indicate that glycolysis in the brain is not as effective as in the heart during cardiac arrest and is not restored following CPB reperfusion.

### Free fatty acid concentration

**Figure 2:**
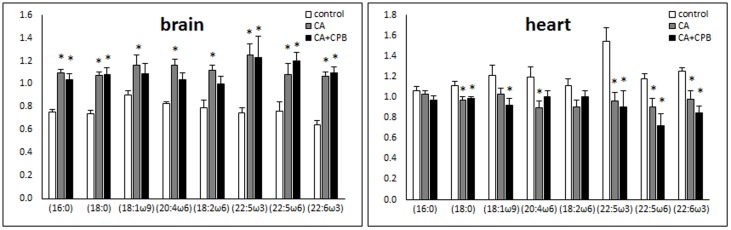
Changes in the relative amounts of free fatty acids in the heart (left) and the brain (right) after 30 min cardiac arrest and 30 min cardiac arrest followed by 60 min CPB (n = 5).

Figure 2 shows changes in the relative content of free fatty acids in heart and brain tissues. In the heart, free fatty acids were decreased following cardiac arrest. There was a tendency that polyunsaturated fatty acids with longer chains were decreased more than saturated or monounsaturated fatty acids with shorter chains. Consistent with the increase in lysophospholipids, free fatty acids were increased in the brain tissue following cardiac arrest. However, unlike heart, there was no difference in the increase between fatty acids with different chain lengths or saturation levels. The amounts of fatty acids did not change significantly during the hour of post-ischemic resuscitation in either tissue.

## Discussion

### Animal model of cardiac arrest

Asphyxia-induced cardiac arrest is an excellent system to study the effect of ischemia in animal models. *In vivo* ischemia not only limits supply of oxygen and substrates but also prevents removal of cellular wastes [28]. As a consequence, ischemia leads to reversible or irreversible organ damage based on the severity of disorders in energy production, electrophysiology, ion homeostasis, and lipid metabolism, etc. Animal models, in which any of these disorders are experimentally manipulated or controlled, can generate artificial factors that interfere with the natural progression of ischemic cellular damage. Other models, such as ventricular fibrillation or KCl-induced cardiac arrest, have a potential to generate additional stress contributing to tissue damage [17], [29]. Asphyxia-induced cardiac arrest is noninvasive and does not require the injection of high concentration of salt, thus allowing the careful examination of ischemia-reperfusion pathology in its native state [18].

After 30 min asphyxial cardiac arrest, animals regained normal sinus rhythm on CPB support, with an average ROSC time of ∼8 min. For an injury model to be useful for developing resuscitation protocols, the injury must be severe enough that traditional resuscitation therapies would be unlikely to succeed while still keeping most organs viable. Viewed in this light, the achievement of ROSC shows that our injury model provides a good basis for investigations into the molecular mechanisms of injury caused by ischemia and reperfusion.

### Metabolic imbalance

Although interpretation on all the metabolites is beyond the scope of this study, some metabolic changes are clearly interpretable and expected under ischemic conditions. From tables 3 and 4, the most noticeable changes are found in metabolites in glycolysis, beta-oxidation, and phospholipid metabolism. The data also indicate alterations in ionic homeostasis in both heart and brain tissues as evidenced by the increased organic osmolytes. Ionic imbalance is an important event for cellular damage and closely related with the lack of ATP and membrane integrity [14]. However, with no direct data regarding cellular ionic or osmotic imbalances, the discussion is focused on metabolites in energy production and phospholipid metabolism.

#### Energy metabolism

ATP is essential for cellular viability during ischemia [28], [30]. Because oxidative phosphorylation is reduced during ischemia, glycolysis becomes the principle method of ATP production in heart and brain tissues [31], [32]. Therefore, the efficiency of glycolysis provides important information on cell viability in these tissues during cardiac arrest.

The levels of glucose and intermediates of glycolysis were significantly decreased, whereas the intermediates of fatty acid oxidation, acyl carnitines and CoAs, were increased (Figure 1 and Table 3). These data indicate the reduced fatty acid oxidation and increased anaerobic glycolysis in heart tissue following cardiac arrest. An inverse change in the same metabolites following CPB indicates that heart resumed aerobic metabolism with return of oxygen supply. This successful conversion between aerobic and anaerobic metabolism shows that the energy generation pathway in heart tissue is not irreparably damaged, if at all.

Table 4 shows that brain tissue consumed ∼90% of glucose during the 30 min cardiac arrest. However, glucose 6-phosphate and fructose-6-phosphate are decreased only by 50% and 25%, respectively. Moreover, 3-phosholgycerate was increased by 78% (Figure 1). These data suggest that the glycolysis in the brain is not as effective as in the heart. Particularly, metabolizing glucose and fructose-6-phosphate without metabolizing intermediates in the later stage of glycolysis will result in no net-generation of ATP. If the other metabolites in the late stage of glycolysis should also accumulate, the decrease in these early metabolites will end up wasting ATP. This unproductive glycolysis may be the reason for the commonly observed rapid ATP depletion in the brain during ischemia [33], and responsible for other cellular disorders resulting in critical brain damage initiated by ATP depletion [34].

#### Phospholipid metabolism

Table 4 and Figure 2 show that the brain not the heart accumulates significant amounts of lysophospholipids and free fatty acids. Lysophospholipids are an intermediate of biosynthesis as well as hydrolysis of membrane phospholipid but, in the context of damage and ischemia, predominantly represent degradation [35]. A decrease in phospholipid content with a concomitant increase in lysophospholipids and free fatty acids is often found in pathological conditions including ischemia [36], [37].

The mechanism for the increase in lysophospholipids and free fatty acids is not clear. The most cited mechanism is increased phospholipase A2-mediated hydrolysis of phospholipid [38], [39]. However, this mechanism does not explain what is found in our study. Activation of phospholipase A2 should accumulate only 1-isomers of lysophospholipids. As shown in Table 4, 2-isomers of lysophospholipids are increased as much as 1-isomers. Furthermore, the data do not support the concept of preferential hydrolysis of certain fatty acids, such as arachidonic acid or docosahexaenoic acid [35], [40]. These results strongly indicate that phospholipase A2 is not solely responsible for the accumulation of lysophospholipids.

Alternatively, decreased reacylation of lysophospholipids rather than increased hydrolysis of phospholipid may be the cause for the accumulation of lysophospholipids. Acylation of lysophospholipids with free fatty acids as substrates requires ATP [41]. As shown by others and suggested in this study, ATP depletes rapidly in brain [42]. The lack of ATP may be the limiting factor preventing reacylation of lysophospholipids, resulting in accumulation of lysophospholipids and free fatty acids. This interpretation also explains the reason for the reduced amount of free fatty acids in heart tissue following cardiac arrest; with the adequately functioning glycolysis, heart generates sufficient amount of ATP for acylation of lysophospholipids, which reduces free fatty acid pool in the absence of influx of fatty acids during ischemia.

A more important aspect of the accumulation of lysophospholipids is the function of this event in the ischemic cascade. Studies have shown deleterious effect of increased lysophospholipids and acyl CoA, which may contribute to cellular damage during ischemia [23], [43], [44]. However, a more critical outcome associated with the increase in lysophospholipids could be the disruption of membrane integrity and membrane function due to depletion of phospholipid concentration [45]. Numerous studies have proposed that impaired phospholipid metabolism is the critical step for irreversible tissue damage [45]–[48]. Our result that 30 min ischemia results in the increase in lysophospholipids and free fatty acids in brain tissue without any observable brain function also supports this hypothesis.

The direct cause for brain damage maybe other metabolic disorders that are also caused by the depletion of ATP. Impaired phospholipid metabolism may just be an indicator that the level of ATP is below the threshold required for cell viability. In any case, the impaired phospholipid metabolism is an important event; identifying the mechanism underlying the increase in lysophospholipids and free fatty acid will help to understand the significance of impaired phospholipid metabolism for progression of ischemic tissue damage in brain.

## Conclusion

Cellular mechanisms underlying ischemic tissue damage involve complex metabolic alterations and identification of the major cause responsible for irreversible tissue damage is highly challenging. Rats subjected to asphyxial cardiac arrest and CPB resuscitation develop biochemical phenotypes of ischemia and unresuscitatable brain damage, the critical pathology of human patients with cardiac arrest, thus this model is a valuable platform to link basic biochemical disorders to organ function in order to design better therapeutic applications for treatment of cardiac arrest.

As shown in this study, the functional and biochemical responses of the heart are different than the responses of the brain to 30 min cardiac arrest followed by 60 min CPB resuscitation. Following CPB resuscitation, the rats exhibit no observable brain function but normal sinus rhythm on CPB support. These physiological outcomes are consistent with the observation that CPB significantly normalizes ischemia-induced metabolic alterations in the heart, but not in the brain. This result suggests that ischemia is the primary cause of brain damage observed following CPB resuscitation. In addition, brain tissue has the increased content of lysophospholipids, whereas this disorder is absent in the heart. This result further supports the notion that impaired phospholipid metabolism is an important phenomena for critical tissue damage during ischemia/reperfusion. However, it is not clear that the observed biochemical and lipid alterations in the brain tissue after CPB are evidence of irreversible brain damage or poor cerebral perfusion during CPB resuscitation.
